# Mesenchymal stromal cell potency to treat acute kidney injury increased by ultrasound‐activated interferon‐γ/interleukin‐10 axis

**DOI:** 10.1111/jcmm.13874

**Published:** 2018-09-14

**Authors:** Scott R. Burks, Matthew E. Nagle, Michele N. Bresler, Saejeong J. Kim, Robert A. Star, Joseph A. Frank

**Affiliations:** ^1^ Frank Laboratory, Radiology and Imaging Sciences Clinical Center, National Institutes of Health Bethesda Maryland; ^2^ Renal Diagnostics and Therapeutics Unit National Institute of Diabetes, Digestive, and Kidney Diseases, National Institutes of Health Bethesda Maryland; ^3^ National Institute of Biomedical Imaging and Bioengineering Bethesda Maryland

**Keywords:** acute kidney injury, cell therapy, cisplatin, focused ultrasound, high intensity focused ultrasound, interferon‐γ, interleukin 10, mesenchymal stem cell, mesenchymal stromal cell, therapeutic ultrasound, ultrasound

## Abstract

Mesenchymal stromal cell (MSC) therapies combined with renal pulsed focused ultrasound (pFUS) pretreatment increase MSC homing and improve cisplatin‐induced acute kidney injury (AKI) better than MSC alone. However, mechanisms underlying improved outcomes remain unknown. We hypothesize pFUS up‐regulates renal interferon‐γ (IFNγ) and stimulates MSC to produce interleukin‐10 (IL‐10) after migrating to kidneys. To demonstrate initially, MSC cultured with IFNγ up‐regulated IL‐10. More MSC‐derived IL‐10 was detected in kidneys when IFNγ‐stimulated MSC were infused and they improved AKI better than unstimulated MSC. Next, IFNγ‐knockout mice with AKI received pFUS+MSC, but MSC‐derived IL‐10 expression and AKI were similar to using MSC alone. AKI in wild‐type mice receiving pFUS and IL‐10‐deficient MSC was also unimproved compared to administering IL‐10‐deficient MSC alone. Indoleamine 2,3‐dioxygenase (IDO), an anti‐inflammatory enzyme up‐regulated in MSC by IFNγ, was up‐regulated during AKI, but was not further elevated in MSC from pFUS‐treated kidneys, suggesting that IDO is not involved in improved AKI healing by pFUS+MSC. These data suggest IFNγ is up‐regulated by pFUS and after i.v.‐infused MSC home to pFUS‐treated kidneys, IFNγ stimulates additional IL‐10 production by MSC to improve AKI. Analogous mechanisms of ultrasound‐treated tissue microenvironments stimulating therapeutic MSC may exist in other pathologies where adjuvant ultrasound techniques are successful.

## 1 INTRODUCTION

Bone marrow mesenchymal stromal cells (MSCs), also known as mesenchymal stem cells, have improved outcomes in numerous disease models.^1^ Transplanted MSCs typically do not engraft in host tissue; rather, the cells persist in situ only temporarily (usually 3‐7 days)^2^, ^3^, ^4^ where they function as local “factories” to secrete paracrine factors into the microenvironment that modulate the immune system and stimulate endogenous regeneration of damaged tissues.^5^ The non‐engrafting immunomodulatory capacity of MSC has been shown effective in disease models where inflammation is critical to pathogenesis, including graft‐versus‐host disease,^6^ ischaemic events,^7^ and autoimmune diseases.^8^

The clinical data regarding MSC therapies are mixed,^9^ leading to wide‐ranging efforts to alter MSC biodistribution following systemic infusion (primarily evade lung clearance^10^) and improve their persistence, potency and function. The simplest modifications to improve efficacy include harvesting MSCs from different source tissues or altering routes of administration, cell doses and dosing schedules. More sophisticated techniques attempt to improve trafficking and homing of systemically infused MSC.^11^, ^12^, ^13^, ^14^, ^15^ Improved targeting of systemically infused MSC has been demonstrated by genetically and/or chemically modifying MSC,^11^, ^12^, ^15^ or physically and/or chemically modifying local host tissue.^13^, ^14^ For example, in vitro preconditioning by culturing with cytokines, growth factors or under hypoxia has improved MSC physiology, biodistribution and potency in vivo by up‐regulating production of specific anti‐inflammatory cytokines or trophic factors.^16^

We have used pulsed focused ultrasound (pFUS) to increase homing of intravenous (i.v.)‐injected MSC to desired locations and shown associations with improved pathology.^17^, ^18^, ^19^, ^20^, ^21^, ^22^ Image‐guided FUS is a noninvasive modality which is a clinically approved treatment for thermal ablation of patients with essential tremor, uterine fibroids and prostate cancer.^23^, ^24^, ^25^ pFUS minimizes heating and allows acoustic mechanical effects to predominate. We have characterized the molecular biological effects of pFUS mechanotransduction in various tissues and show that pFUS stimulates local release of cytokines, chemokines, and trophic factors (CCTF) into the microenvironment and up‐regulates cell adhesion molecules on endothelial cells.^17^, ^18^ This local molecular biological response to pFUS represents chemoattractants that further increase homing of systemically infused MSC to sonicated muscle and kidneys.

In mouse models of cisplatin‐induced acute kidney injury (AKI), i.v.‐infused MSC have some intrinsic capability to home to kidneys and improve AKI.^26^ We further improved this approach by administering pFUS to kidneys prior to human MSC infusions, which led to increased MSC homing and better‐improved AKI compared to MSC infusions without pFUS pretreatment.^17^ While outcomes were greatly improved, the mechanism behind the improved therapy was unknown. We initially attributed improved outcomes to increased numbers of MSC homing to pFUS‐treated tissue. However, a disproportionally high expression of MSC‐produced human interleukin‐10 (IL‐10) was present in pFUS‐treated kidneys, suggesting that homing to pFUS‐treated kidneys elicited different MSC physiology compared to homing to unsonicated kidneys.

This study investigated whether MSC‐derived IL‐10 was critical for improved AKI outcomes following renal pFUS and what stimulated its increased expression. IL‐10 is an anti‐inflammatory cytokine that has been demonstrated to directly improve both cisplatin‐ and ischaemia/reperfusion‐induced AKI.^27^ We previously observed that pFUS alone stimulated AKI kidneys to increase the production of interferon (IFN)γ.^17^ The potential involvement of IFNγ was intriguing, as culturing MSCs with pro‐inflammatory cytokines “primes” MSCs to produce more anti‐inflammatory factors and become a more effective therapeutic cell factory.^28^, ^29^ We initially performed a simple in vitro preconditioning study of MSC by culturing them with recombinant IFNγ. We then employed combinations of transgenic IFNγ‐deficient mice and IL‐10‐deficient MSCs to selectively probe the molecular mechanisms behind pFUS improving MSC therapy for AKI in vivo.

## 2 MATERIALS AND METHODS

### 2.1 Animals and AKI

Animal studies were approved by the National Institutes of Health Clinical Center Animal Care and Use Committee. All procedures and experiments were performed in accordance with relevant guidelines and regulations. Female C3H or B6.129S7‐*Ifngr1* ^*tm1Agt*^/J mice (The Jackson Laboratory, Bar Harbor, ME) were used for this study. All animals were aged 8‐10 weeks during experiments. Mice had free access to food and water except for 12 hours prior to cisplatin injections. AKI was induced by intraperitoneal (i.p.) injection of cisplatin (15 mg/kg) (Fresenius Kabi USA, Lake Zurich, IL) on day 0 (D0) and free access to food and water was restored immediately after injection.

### 2.2 Pulsed focused ultrasound

Twenty‐four hours after cisplatin injection (D1), pFUS was delivered under ultrasound imaging guidance using a VIFU 2000 and E‐Cube 12 (Alpinion, Bothell, WA). Mice were anaesthetized with isoflurane (2.5% in 100% O_2_) and kidneys were sonicated using the following parameters: 1 MHz ultrasound, 4 MPa peak negative pressure, 10 ms pulse length, 5 Hz pulse repetition frequency, and 100 pulses per site. Sonication points were spaced 2 mm apart and the number of points varied depending on the size of the kidney (most kidneys were entirely treated using 9–12 loci). pFUS treatment alone did not alter AKI outcomes (Figure S3). Mice that were treated to measure MSC homing were given unilateral pFUS and the contralateral kidney was used as the untreated internal control. Mice in treatment groups to measure AKI outcomes were given kidney pFUS bilaterally. Groups that were not treated with pFUS received sham exposures (transducer power = 0 W) and were considered controls.

### 2.3 MSC culture and infusions

Human MSCs were donated to the NIH Center for Bone Marrow Stromal Cell Transplantation under the clinical trial NCT01071577 (http://www.clinicaltrials.gov), which was approved by the NIH Clinical Center IRB and included informed consent from donors. MSCs were cultured in α‐minimum essential medium supplemented with foetal bovine serum (20%). MSCs for this study were previously characterized for cell surface marker expression^17^ and all experiments employed MSC at passage number five or less. MSC were cultured in 175 cm^2^ flasks and were allowed to reach ~80% confluence before use. In some experiments, MSCs were treated in vitro with 250 U/mL of recombinant murine IFNγ (Peprotech, Rocky Hill, NJ) in complete medium for 24 hours. For infusions into mice, MSCs were harvested using a 0.5% trypsin solution containing 1 mmol/L ethylene diamine tetraacetic acid (EDTA) and resuspended to 10^7^ cells/mL in Hank's balanced salt solution without divalent ions that contained 10 U/mL sodium heparin. Approximately 4 hours post‐pFUS, mice were given an i.v. injection of sodium nitroprusside into the lateral tail vein (1 mg/kg in 0.9% saline).^30^ Immediately after sodium nitroprusside, 10^6^ MSC (in 100 μL) were infused into the opposite lateral tail vein. Groups of mice that were not treated with MSC did receive injections of sodium nitroprusside and saline as controls.

### 2.4 IL‐10 knockdown in MSC

IL‐10 expression was knocked down in MSC using multiple human‐IL‐10‐specific siRNA sequences (Cat. No. sc‐39634; Santa Cruz Biotechnology, Santa Cruz, CA). MSCs were plated at a density of 5.6 × 10^4^ MSC/cm^2^ 24 hours before siRNA treatment. siRNA was added to a lipid‐based transfection agent (Santa Cruz) and allowed to incubate at room temperature for 20 minutes before being diluted in siRNA transfection medium according to the manufacturer's protocol (Santa Cruz). siRNA was added to cells at 150 pg/10^6^ MSC for 6 hours. siRNA‐containing medium was then diluted 1:2 with complete medium and cells were incubated another 24 hours. Cultures were then aspirated and MSCs were either prepared for animal infusions (see above) or incubated in cell lysis buffer (Cell Signaling Technology, Danvers, MA) containing a protease inhibitor cocktail (Santa Cruz) for IL‐10 expression analysis by western blot (see below). Control siRNA sequences (cat. No. sc‐37007; Santa Cruz) were transfected into MSC using the same protocol.

### 2.5 Tissue harvesting and sample processing

Mice that were treated for molecular analyses and quantification of MSC homing were killed on D2 (24 hours post‐MSC infusion; 48 hours post‐cisplatin). Mice that were treated to assess AKI outcomes by histology and renal function were killed on D4 (72 hours post‐MSC infusion; 96 hours post‐cisplatin). Kidneys were either frozen in liquid N_2_ or fixed in 4% paraformaldehyde (PFA) for 24 hours. Serum was isolated by centrifugation using EDTA as an anti‐coagulant and stored at −80°C. Fixed kidney tissue was embedded in paraffin and sectioned at 5 μm. Frozen kidneys were mechanically homogenized in cell lysis buffer (Cell Signaling Technology) containing protease inhibitors (Santa Cruz) using a Bead Beater (BioSpec, Bartlesville, OK). Homogenized samples were centrifuged for 30 minutes at 4°C and the supernatants were frozen at −80°C for later use. Total protein concentration of homogenized samples was determined spectrophotometrically using a bicinchoninic acid assay.

### 2.6 Renal function measurements

Renal function was measured using assay kits (Sigma Aldrich, St. Louis, MO). Blood urea nitrogen (BUN) was measured in serum spectrophotometrically following degradation by urease. Serum creatinine (SCr) was measured fluorometrically following degradation by creatininase; this assay reports results similar to high‐pressure liquid chromatography methods.^31^

### 2.7 Molecular analyses

Human and mouse cytokines were measured in kidney homogenates using species‐specific enzyme‐linked immunosorbent assays (ELISA) for human IL‐10 (Cat# D1000B) human indoleamine 2,3‐dioxygenase (IDO; Cat# DY6030‐05), murine IL‐10 (Cat# M1000B) and murine IFNγ (Cat# MIF00) (R&D Systems, Minneapolis, MN). Antibodies were demonstrated by the manufacturer to be free of interspecies cross‐reactivity (ie. the human and mouse IL‐10 antibodies detected only IL‐10 from that species). ELISAs were loaded with tissue homogenates at a concentration of 2 mg/mL and developed according to manufacturer protocols. Western blotting was performed by loading 20 μg of total protein were resolved by sodium dodecyl sulphate‐polyacrylamide gel electrophoresis on a 4%‐12% polyacrylamide gel under reduced conditions. Proteins were transferred onto nitrocellulose membranes, blocked in 5% bovine serum albumin and hybridized with primary antibodies overnight at 4°C. Primary antibodies (dilutions in parentheses) included human IL‐10 (1:500), murine IL‐10 (1:500) (eBioscience, San Diego, CA), or β‐actin (1:5000) (Abcam, Cambridge, MA). Species‐appropriate and fluorescently labelled secondary antibodies (dilutions ranging from 1:1000 to 1:10000) (Abcam) were incubated for 1 hour at room temperature. Blots were imaged using a ChemiDoc MP system (Bio‐Rad, Hercules, CA).

### 2.8 Histological analyses

Tissue sections were deparaffinized in xylenes and rehydrated. Antigen retrieval was performed with 10 mmol/L citrate buffer (pH 6.0) and sections were blocked with SuperBlock (ThermoFisher Scientific, Waltham, MA). Primary antibodies against human mitochondria (1:200), murine KIM‐1 (1:500) (Abcam) or human IL‐10 (1:200) (eBioscience) were incubated on sections overnight at 4°C. For human mitochondria, an Alexa Fluor 647‐labelled secondary antibody was hybridized on sections for 2 hours at room temperature. We have previously characterized this antibody to specifically detect human MSC in kidneys.^21^ For KIM‐1, a horseradish peroxidase conjugated secondary antibody and reacted with 3,3′‐diaminobenzidine (DAB). Slides were coverslipped using ProLong Diamond anti‐fade mounting medium containing 2‐(4‐amidinophenyl)‐1H‐indole‐6‐carboxamidine (DAPI) (Life Technologies, Carlsbad, CA) or Permount mounting medium. Control sections were stained with species‐specific IgG as isotype controls. Cell death was measured using an In Situ Cell Death Detection, Fluorescein kit (Roche, Basel, Switzerland). Examples of KIM‐1 and TUNEL staining in healthy mice are shown in Figure S2.

### 2.9 Microscopy

Slides were scanned using an Aperio CSO brightfield slide scanner (Leica Biosystems, Buffalo Grove, IL) or an Aperio‐FL epifluorescence slide scanner. Both scanners were equipped with 20× PlanApo objectives (numerical aperture = 0.75). Images were segmented using ImageJ (National Institutes of Health, Bethesda, MD) and total KIM‐1 signal was quantified, corrected to isotype background signal and normalized to signal in healthy kidneys. MSCs were quantified by manually counting 10 fields‐of‐view per slide from three slides per animal. Reviewers were blinded to conditions and counts were background corrected by subtracting counts in isotype‐stained slides.

### 2.10 Statistical analyses

Data are presented as the mean ± standard deviation. Pairwise comparisons were made using Student's *t* tests and multiple comparisons were made using one‐way analysis of variance using Prism (v6 Graphpad Inc. La Jolla, CA). All statistical tests were two‐sided and *P* values <0.05 were considered significant.

## 3 RESULTS

### 3.1 Stimulating human MSC with murine IFNγ increases MSC IL‐10 production in vitro, and improves AKI better than unstimulated MSC

**Figure 1:**
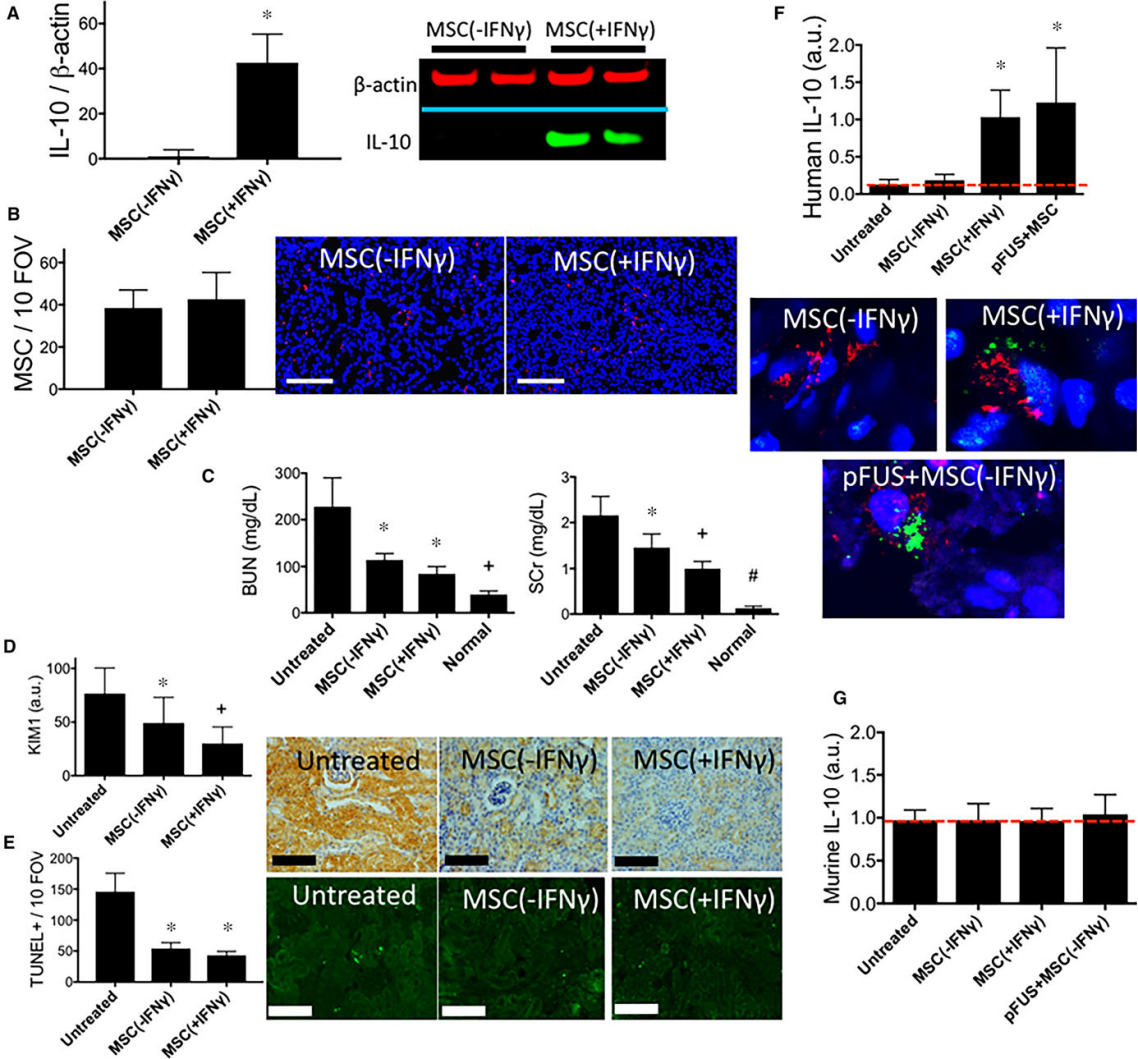
Culturing mesenchymal stromal cell (MSCs) with IFNγ in vitro increases IL‐10 expression and increases their potency to treat AKI. (A) Western blotting demonstrates that in vitro exposure of human MSCs with recombinant murine IFNγ (250 U/mL) for 24 h increases MSC expression of IL‐10 (n = 6 per group, *P* < 0.05). (B) Either IFNγ‐treated (+IFNγ) MSC or normal control MSC (−IFNγ) were i.v infused into mice (10^6^ per mouse) and human mitochondria were detected in mouse kidneys by IHC (MSCs = red; nuclei = blue). No statistically significant difference in the number of MSC homing to kidneys was observed with IFNγ treatment compared to control MSC (*P* > 0.05). (C) Levels of BUN and SCr were significantly (*P* < 0.05) decreased by infusing IFNγ‐treated MSC compared to control MSC. A statistically insignificant trend (*P* = 0.051) of (D) decreased KIM‐1 was observed following infusion of IFNγ‐treated MSC. (E) Significantly fewer TUNEL+ cells (*P* < 0.05) were also observed following infusion of IFNγ‐treated MSC (scale bars = 100 μm). (F) ELISA from kidney homogenates revealed greater human IL‐10 levels in kidneys when MSC were either pretreated with IFNγ in vitro, or when unstimulated MSC were infused into mice which received pretreatment with kidney pFUS. Immunostaining for human mitochondria (red) to identify individual MSC and human IL‐10 (green) revealed greater IL‐10 expression in IFNγ‐pretreated MSC or MSC infused into mice pretreated with pFUS compared to mice receiving unstimulated MSC with non‐pFUS‐treated AKI kidneys. (G) None of the treatment groups altered murine IL‐10 expression in kidneys

Preconditioning human MSC by culturing with recombinant murine IFNγ (250 U/mL) for 24 hours significantly increased (*P* < 0.05) in vitro production of IL‐10 (Figure 1A, Figure S1). MSCs preconditioned with IFNγ (+IFNγ) or cultured under normal conditions (−IFNγ) were intravenously (i.v.) injected into wild‐type (WT) mice with AKI (n = 6/group). One day later, mice were killed and kidneys immunostained for human mitochondria to detect MSC (Figure 1B). IFNγ preconditioning did not statistically change the number of MSC homing to AKI kidneys. Other groups of mice given either type of MSC (n = 6) were killed 3 days post‐infusion. Small but statistically significant (*P* < 0.05) improvements in BUN and SCr were observed in mice that received the +IFNγ MSC compared to −IFNγ MSC mice (Figure 1C) and there was also a trend for lower KIM‐1 expression (*P* = 0.051) (Figure 1D) following +IFNγ MSC treatment. Moreover, there are fewer TUNEL‐positive cells (*P* < 0.05) (Figure 1E). Figure 1F shows significantly more human (MSC‐produced) IL‐10 was detected in AKI kidneys that received +IFNγ MSC compared to −IFNγ MSC infusions. Furthermore, immunostaining for IL‐10 and human mitochondria revealed increased IL‐10 expression in MSC that were stimulated with IFNγ (+IFNγ MSC) in vitro or unstimulated MSCs (−IFNγ MSC+pFUS) that were infused into AKI mice which received pFUS prior to infusion. Immunostaining for IL‐10 revealed no expression in unstimulated MSCs that were infused in to AKI mice which did not receive pFUS (−IFNγ MSC alone). None of these treatment groups altered expression of murine renal IL‐10 (Figure 1G).

### 3.2 Additional protection afforded by pFUS+MSC is absent in IFNγ knock‐out mice

**Figure 2:**
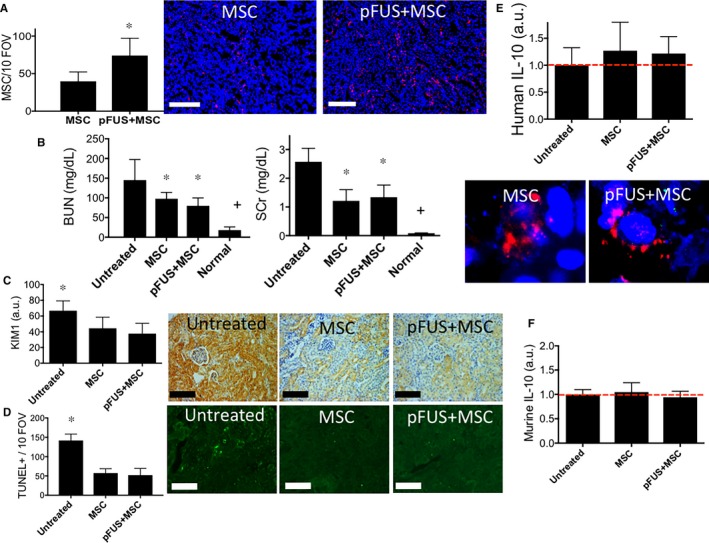
Mesenchymal stromal cells (MSCs) infused into INFγ‐KO mice following pFUS to kidneys do not express more IL‐10 and do not further improve AKI compared to MSC infusions alone. INFγ‐KO mice with AKI were given intravenous infusions of 10^6^ human MSC with or without pFUS (n = 6 mice for all experimental groups). (A) pFUS significantly increased MSC homing to INFγ‐KO AKI kidneys (*P* < 0.05). Fluorescence IHC for human mitochondria is shown to the right (scale bar = 100 μm; MSC = red; nuclei = blue). (B) Renal function (BUN and SCr clearance) in INFγ‐KO mice was significantly improved by MSC alone (*P* < 0.05 compared to untreated controls), but additional significant reductions were not observed when mice were treated with pFUS+MSC (*P* > 0.05 compared to MSC alone). (C) Renal KIM‐1 expression and (D) number of TUNEL+ cells in INFγ‐KO mice are significantly improved by infusion of MSC alone (*P* < 0.05 compared to untreated controls), but not further reduced (*P* > 0.05 compared to MSC alone) with pFUS+MSCs (all scale bars = 100 μm). (E) ELISA showing human IL‐10 undetectable in kidneys from INFγ‐KO mice that received pFUS+MSC or kidneys from INFγ‐KO mice that received MSC alone. For reference, kidneys from INFγ‐KO mice (AKI, but no MSC or pFUS) are shown with the red line to indicate assay background levels. IHC staining of human mitochondria (red) and human IL‐10 (green) revealed no increased IL‐10 expression in MSC following infusion into INFγ‐KO mice with pFUS‐pretreated kidneys. (F) Murine IL‐10 was levels were unchanged in INFγ‐KO mice with AKI that received MSC alone or pFUS+MSC compared to untreated AKI mice. Groups with identical symbols are statistically similar and are statistically different from groups with different symbols

IFNγ‐KO mice (n = 6/group) were given cisplatin and then divided into three groups; cisplatin alone (untreated control); MSC alone; and pFUS+MSC. Healthy IFNγ‐KO mice served as normal controls and were administered saline intraperitoneally (i.p.). pFUS alone was demonstrated not to alter AKI progression or outcomes^17^ and therefore those controls were omitted from this and subsequent experiments. IFNγ was not detected in the kidneys of KO mice with or without targeted pFUS treatment (Figure S4). Nearly twice as many human MSC homed to pFUS‐treated kidneys of IFNγ‐KO mice compared to kidneys of IFNγ‐KO mice that did not receive pFUS (*P* < 0.001) (Figure 2A). Cohorts of IFNγ‐KO mice that received MSC alone or pFUS+MSC demonstrated significant improvements in BUN and SCr in (*P* < 0.05) compared to untreated KO controls. However, there was no difference in BUN and SCr (*P* > 0.05) between the MSC alone and pFUS+MSC treated IFNγ‐KO mice (Figure 2B). A similar failure between the two treatment cohorts to further reduce the expression of KIM‐1 (Figure 2C), or TUNEL‐positive cells (Figure 2D). Human IL‐10 was essentially undetectable in the three cohorts (Figure 2E). Lastly, murine IL‐10 did not change (*P* > 0.05) between untreated AKI controls and either of the treatment groups (MSC with or without pFUS) (Figure 2F).

### 3.3 Additional protection afforded by pFUS+MSCs is absent when MSC are IL‐10‐deficient

**Figure 3:**
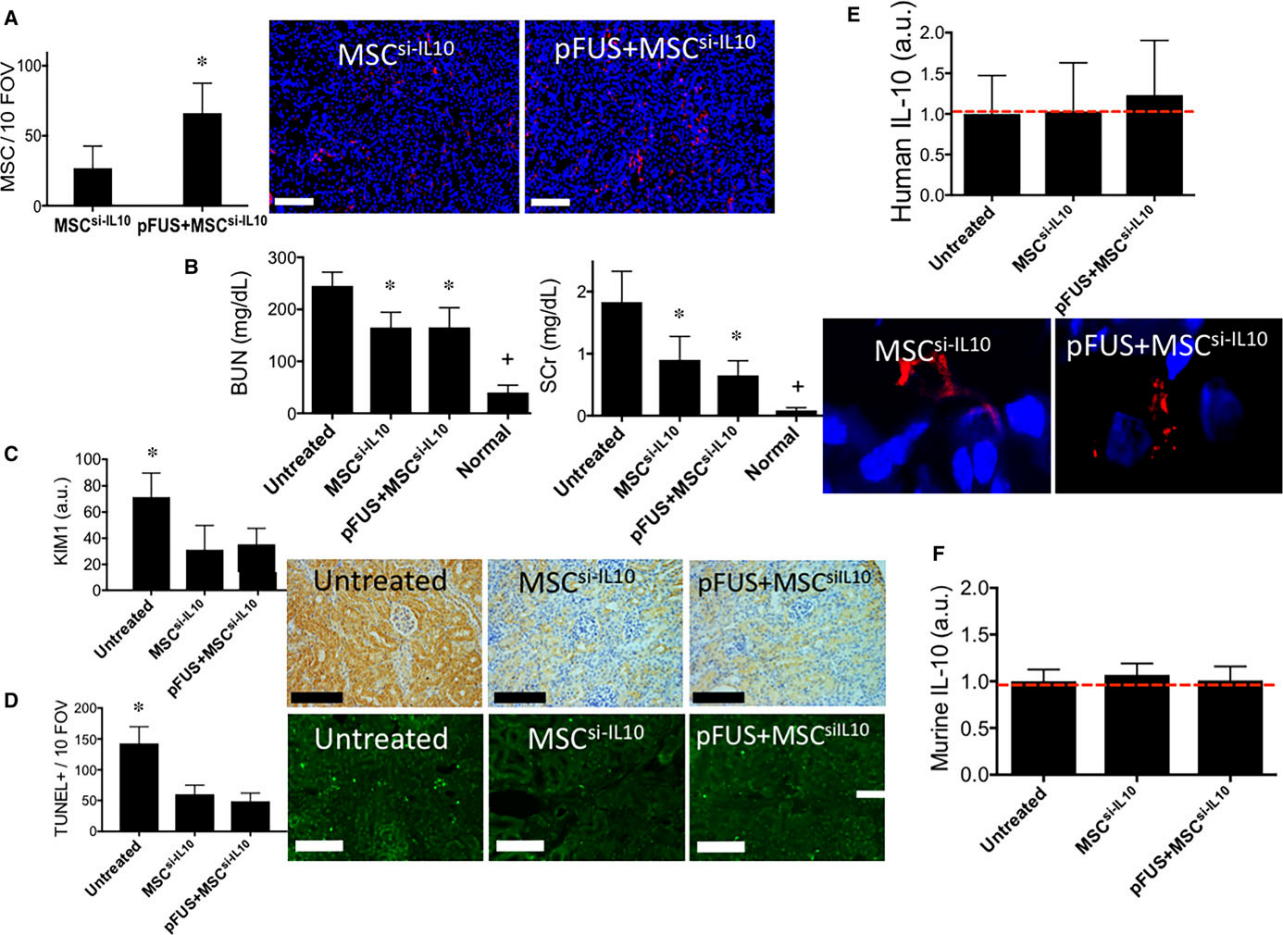
IL‐10‐silenced mesenchymal stromal cells (MSCs) infused into wild‐type C3H mice following pFUS to kidneys do not increase IL‐10 expression or improve AKI outcomes compared to infusions of IL‐10‐silenced MSCs alone. C3H mice with AKI were given i v infusions of 10^6^ IL‐10‐silenced human MSC (MSC ^si‐^ ^IL^ ^10^) with or without pFUS (n = 6 mice for all experimental groups). (A) pFUS significantly increased MSC ^si‐^ ^IL^ ^10^ homing to AKI kidneys (*P* < 0.05). Fluorescence IHC for human mitochondria is shown to the right (scale bar = 100 μm; MSC = red; nuclei = blue). (B) Renal function (BUN and SCr clearance) in C3H mice was significantly improved by MSC ^si‐^ ^IL^ ^10^ alone (*P* < 0.05 compared to untreated controls), but additional significant reductions were not observed when mice were treated with pFUS+MSC ^si‐^ ^IL^ ^10^ (*P* > 0.05 compared to MSC alone). (C) Renal KIM‐1 expression, D) TUNEL+ cells during AKI in C3H mice are significantly improved by infusion of MSC ^si‐^ ^IL^ ^10^ alone (*P* < 0.05 compared to untreated controls), but not further reduced (*P* > 0.05 compared to MSC alone) with pFUS+MSC ^si‐^ ^IL^ ^10^. Representative IHC shown at right (scale bar = 100 μm). (E) Human IL‐10 expression remained undetectable in kidneys from mice that received pFUS+MSC ^si‐^ ^IL^ ^10^ or MSC ^si‐^ ^IL^ ^10^ alone. For reference, kidneys from untreated C3H mice (AKI, but no MSC or pFUS) are shown with the red line to indicate assay background levels. IHC staining of human mitochondria (red) and human IL‐10 (green) revealed no increased IL‐10 expression in MSC ^si‐^ ^IL^ ^10^ following infusion into mice with pFUS‐pretreated kidneys. F) Murine IL‐10 levels were unchanged in C3H mice with AKI that received MSC alone or pFUS+MSC compared to untreated AKI mice. Groups with identical symbols are statistically similar and are statistically different from groups with different symbols

To determine if MSC production of IL‐10 is mechanistically important, we used siRNA to silence IL‐10 expression in human MSC. MSC transfected with siRNA sequences against IL‐10 did not up‐regulate IL‐10 when cultured with IFNγ, whereas IFNγ was able to induce IL‐10 expression in MSCs transfected with scramble‐siRNA sequences (Figure S5). Renal function (BUN and SCr) was not significantly different whether WT C3H mice were given infusions with normal MSC, or IL‐10‐siRNA‐transfected MSC (MSC^si‐IL10^) (Figure 3B). C3H mice were given cisplatin, then randomized into three groups: untreated controls; MSC^si‐IL10^ alone; or pFUS+MSC^si‐IL10^. Nearly twice as many MSC^si‐IL10^ homed to pFUS‐treated compared to unsonicated kidneys (Figure 3A). Infusions of MSC ^si‐IL10^ alone did significantly improve renal function compared to untreated AKI mice (*P* < 0.05). The pFUS+MSC^si‐IL10^ did not further improve renal function (*P* > 0.05) and had compatible effects to the MSC^si‐IL10^ alone cohort (Figure 3B). Similarly, both cell treatment cohorts significantly reduced KIM‐1 expression (Figure 3C) and TUNEL‐positive cells (Figure 3D), but the cohorts receiving MSC^si‐IL10^ alone or in combination with pFUS were not significantly different from each other (all *P* > 0.05). IL‐10 was not detected in kidneys from cisplatin‐treated C3H mice that received infusions of MSC^si‐IL10^ or pFUS+MSC^si‐IL10^ (*P* > 0.05) when compared to untreated AKI controls (Figure 3E). Murine IL‐10 did not change between WT AKI mice that received either treatment or those that served as untreated controls (Figure 3F).

### 3.4 IDO expression is stimulated in MSC by AKI‐associated IFNγ, but further increasing IFNγ levels with pFUS does not yield similar increases in IDO

**Figure 4:**
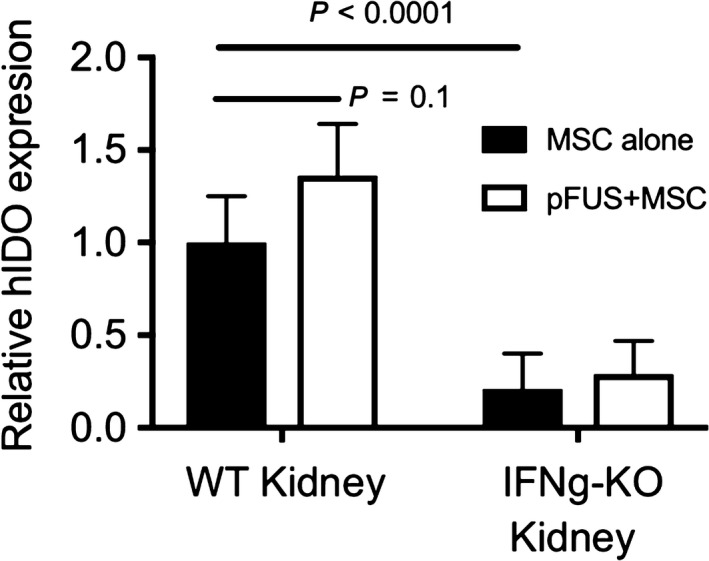
IDO expression is up‐regulated in mesenchymal stromal cells (MSC) that home to AKI kidneys, but the increased renal IFNγ following pFUS does not further up‐regulate IDO expression. Kidney homogenates from WT and INFγ‐KO mice that had AKI and received MSC treatment with or without pFUS were assayed for IDO expression by ELISA. For, mice that received MSC without pFUS, greater IDO expression was observed in WT mice compared to INFγ‐KO mice (*P* < 0.0001). This represents IDO activation through AKI‐expressed INFγ‐KO. However, when WT mice received pFUS to further up‐regulate INFγ, on a statistically insignificant trend for additional IDO expression was observed (*P* = 0.1). Displayed IDO quantities were background corrected using AKI kidneys from mice that did not receive MSC treatment and normalized to relative MSC quantities present in kidneys with and without pFUS

We also investigated the role IDO, an important immuno‐regulator of human MSC that is also stimulated by IFNγ.^32^ ELISAs measured IDO expression in AKI kidney homogenates from WT or IFNγ‐KO mice that received either MSC alone or MSC+pFUS (Figure 4). IDO expression was expressed as a function of the number of MSC homing to kidneys in each condition (ie. to control for more MSC observed after pFUS). Per MSC in each kidney type, IDO was ~5‐fold greater in WT AKI mice receiving MSC alone compared to IFNγ‐KO mice receiving MSC alone (*P* < 0.0001). This suggests that IFNγ that is expressed during AKI stimulates some IDO expression in MSC that home to kidneys. However, in WT kidneys that received pFUS+MSC, there was a small (~1.3‐fold increase) and statistically insignificant increase (*P* = 0.1) in IDO observed as a function of pFUS. As expected, pFUS did not change the limited expression of IDO in MSC that homed to kidneys in IFNγ‐KO mice.

## 4 DISCUSSION

**Figure 5:**
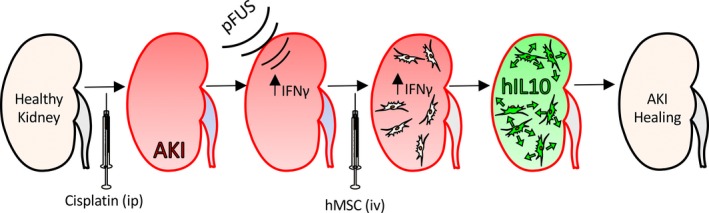
Schematic detailing molecular mechanisms behind combination pFUS and mesenchymal stromal cells (MSC) therapy in cisplatin AKI. Following cisplatin infusion and establishment of AKI, pFUS up‐regulates renal IFNγ which stimulates subsequently infused MSC to up‐regulate IL‐10 and promote AKI healing

The study demonstrated that altering the host microenvironment with noninvasive pFUS potentiates therapeutic MSC to further improve pathological outcomes. Combining renal pFUS with MSC infusion during cisplatin AKI activates a cytokine axis involving renal IFNγ and MSC IL‐10. pFUS exposure to kidneys elevates renal IFNγ levels which directly condition subsequently infused MSC to produce more IL‐10 after they home to treated kidneys (see Figure 5 for schematic). This cytokine axis is responsible for the synergy between pFUS pretreatment and MSC compared to infusing MSC alone.

We have extensively investigated the molecular effects of pFUS^18^, ^19^, ^20^, ^21^, ^33^ and employed pFUS to improve MSC tropism and outcomes in several disease models.^17^, ^20^, ^34^ When MSCs are not stimulated by the pFUS‐altered renal microenvironment (ie. in the IFNγ‐KO mouse or using IL‐10‐silenced MSC), despite increased tropism in the pFUS‐treated groups, the additional cells did not improve AKI outcomes. We demonstrated that the IFNγ/IL‐10 cytokine axis is essential for improved AKI outcomes, but that increased MSC tropism occurs through additional pFUS‐related molecular mechanisms that involve tumour necrosis factor, IL‐1α and cyclooxygenase‐2 signalling.^35^

IL‐10 is an important therapeutic target in AKI. IL‐10 reduces injury in several AKI models^27^, ^36^, ^37^, ^38^ and mediates an MSC effect in sepsis and AKI.^39^ Xenotransplantation of human MSC in mice renders MSC‐derived IL‐10 (human) distinguishable from host‐derived IL‐10 (mouse) and reveals that the additional IL‐10 produced by MSC is necessary for the improved AKI outcomes with pFUS. Murine IL‐10 levels were unchanged by either treatment (MSC or pFUS+MSC) compared to untreated AKI controls. Moreover, we have previously demonstrated that pFUS alone (no MSC infusions) did not alter endogenous murine IL‐10 levels or AKI outcomes despite the numerous other molecular changes associated with sonication.^17^ It is not immediately clear why pFUS‐associated molecular changes alone were insufficient to influence AKI outcomes, or if they also induce homing of endogenous MSC, but AKI outcomes are only changed by infusions of exogenous MSC. While MSC‐produced IL‐10 is critical to improve MSC therapeutic efficacy using pFUS, the exact function of MSC‐derived IL‐10 will require additional investigation. For example, understanding the temporal expression profile of IL‐10 will be critical to optimizing this therapeutic approach.

Various in vitro preconditioning techniques have been used to alter the functional responsiveness of MSC by increasing their potency. Preconditioning MSC with growth factors or pro‐inflammatory agents can enhance the paracrine effects and the production of anti‐inflammatory or trophic factors following implantation within tissues.^40^, ^41^ MSC cultured under hypoxic conditions exhibit improved survival and therapeutic responsiveness.^42^, ^43^ While this study elucidates the mechanism behind combination therapy of pFUS and MSCs for cisplatin‐induced AKI, there are wide‐ranging implications for this noninvasive approach to improve other cellular therapies used in regenerative medicine. For example, we demonstrated a potential link between pFUS and cell potency in a model of critical limb ischaemia.^34^ To our knowledge, pFUS represents the first demonstration of an in vivo stem cell conditioning tool. Other approaches involve exogenous manipulation of cell products to enhance MSC potency. Image‐guided pFUS has been clinically approved to treat a variety of pathologies. US‐guided renal pFUS could be performed at the bedside, possibly making the approach more feasible and widely available pending appropriate clinical approvals in the treatment of AKI.

Mesenchymal stromal cells have received substantial attention to treat AKI experimentally and a phase 1 clinical trial to treat cisplatin‐induced nephropathy is ongoing (NCT01275612; clinicaltrials.gov). In contrast, the introduction of ultrasound‐based techniques as possible adjuvants to MSC therapies have received limited investigation. A previous study demonstrated that non‐thermal ultrasound‐targeted destruction of microbubbles contrast agents in the kidney enhanced homing of MSC and improved renal function in rats following mercuric chloride injury.^44^ In that study, ultrasound interaction with intravascular microbubbles generated inertial cavitation (destruction of microbubbles) and sent pressure waves (ie. shockwaves) into the parenchyma. The pFUS sonications described here have been applied to renal and muscle tissue without significant cellular damage—a likely necessity for regenerative medicine approaches.^17^, ^18^, ^19^, ^20^, ^21^, ^22^, ^34^ Interestingly, it has been reported that diagnostic ultrasound to the spleen stimulates cholinergic anti‐inflammatory pathways through haematopoietic nicotinic acetylcholine receptors to reduce inflammation and improve the specific duration of ischaemic reperfusion induced AKI.^45^, ^46^

Other mechanism(s) besides the renal‐IFNγ/MSC‐IL‐10 are responsible for the recovery in AKI when infusing MSC alone and presumably still ongoing when coupling MSC with pFUS. These mechanisms might include, for example: (a) MSC secreting other CCTF into the AKI microenvironment such as insulin growth factor‐1, placenta derived growth factor or hepatocyte growth factor that have been shown to stimulate renal perfusion and tubular cell proliferation^47^; (b) the release of microvesicles from the homed MSC, which contain CCTF and nucleic acids that can accelerate cellular recovery^48^; (c) MSC modulate the apoptotic responses of tubular cells by up‐regulating B‐cell lymphoma 2 (Bcl‐2) and downregulating Bcl‐2‐associated X (BAX)^49^; or (d) MSC modulate the immune system to reduce inflammation and promote healing (eg. through IDO signalling). It is important to note that IDO expression was greater in MSC from WT AKI kidneys compared to IFNγ‐KO kidneys. Presumably IDO up‐regulation occurs through exposure to IFNγ that is expressed as part of the AKI processes. This is difficult to say with certainty since AKI in the IFNγ‐KO mice might not be the same disease despite similar levels of injury being reported between KO and WT mice.^50^ Additionally, the cohorts in this study did not respond to cisplatin with as severe of AKI as previously observed.^17^ However, it is clear that further increasing renal IFNγ concentrations with pFUS in WT mice did not yield analogous elevations in MSC IDO. Possible explanations for this include IDO expression being maximized by endogenous AKI‐associated IFNγ, and therefore MSCs are unresponsive to additional IFNγ produced by pFUS. Alternatively, a single MSC donor was used for all AKI studies in our lab and it is possible that this particular donor's IDO responsiveness is less than that of other MSC populations. We were surprised by the IDO findings in this study, but we recognize the potent immunomodulatory capacity of IDO and its potential as an additional target to be up‐regulated by pFUS preconditioning. Therefore, as this approach to MSC therapy is expanded to other pathologies, IDO should certainly be investigated as a mediator of improved cell potency with pFUS.

In conclusion, pFUS increased the therapeutic potency of MSC to reduce tubular injury and improve renal function in a cisplatin AKI model though pFUS‐induced up‐regulation of renal INFγ that stimulated additional MSC production of IL‐10; this pathway is unrelated to the increase of MSC homing to pFUS‐treated AKI kidneys. Further investigations are needed to determine if other AKI models or inflammatory diseases will be treatable by similar regenerative medicine approaches that use pFUS to precondition the diseased tissue. pFUS represents a noninvasive and clinically translatable technique to increase both MSC tropism and potency and can be investigated in numerous other pathologies to improve the effectiveness of cellular therapy approaches in regenerative medicine.

## AUTHOR CONTRIBUTIONS

All authors read and approved the content of the manuscript. SRB: Designed experiments, performed experiments, analysed data, wrote manuscript. MEN and MNB: Performed experiments, analysed data. SJK: Designed and performed experiments. RAS: Analysed data, wrote manuscript. JAF: Designed experiments, wrote manuscript.

## ACKNOWLEDGEMENTS

This study was funded by the Intramural Research Program at the National Institutes of Health Clinical Center, the National Institute of Biomedical Imaging and Bioengineering and the National Institute of Diabetes, Digestive Disease, and Kidney Diseases.

## CONFLICTS OF INTEREST

The authors confirm there are no conflicts of interest.
