# Supplementary material for: Mesenchymal stromal cell potency to treat acute kidney injury increased by ultrasound‐activated interferon‐γ/interleukin‐10 axis
Source: J Cell Mol Med. 2018 Sep 14;22(12):6015–25. doi: 10.1111/jcmm.13874 (PMC6237567; doi:10.1111/jcmm.13874)
Supplement: Supplementary file 1 [file JCMM-22-6015-s001.docx]

Supplemental Data for:

Mesenchymal stromal cell potency to treat acute kidney injury increased by ultrasound-activated interferon-γ/interluekin-10 axis

Scott R. Burks, Ph.D.^1*^, Matthew E. Nagle, B.S.^1^, Michele N. Bresler, B.S.^1^, Saejeong J. Kim, Ph.D.^1^, Robert A. Star, M.D.^2^, and Joseph A. Frank, M.S., M.D. ^1,3^

^1^Frank Laboratory, Radiology and Imaging Sciences, Clinical Center, National Institutes of Health, Bethesda, MD 20892

^2^Renal Diagnostics and Therapeutics Unit, National Institutes of Diabetes, Digestive, and Kidney Diseases, National Institutes of Health, Bethesda MD 20892

^3^National Institute of Biomedical Imaging and Bioengineering, Bethesda, MD 20892

Short title: Renal US pretreatment improves therapeutic MSC efficacy

Address correspondence to Scott R. Burks, Ph.D.; [scott.burks@nih.gov](mailto:scott.burks@nih.gov)

Keywords: ultrasound, focused ultrasound, high intensity focused ultrasound, cell therapy, mesenchymal stromal cell, mesenchymal stem cell, cisplatin, acute kidney injury, interleukin 10, interferon-γ, cell potency


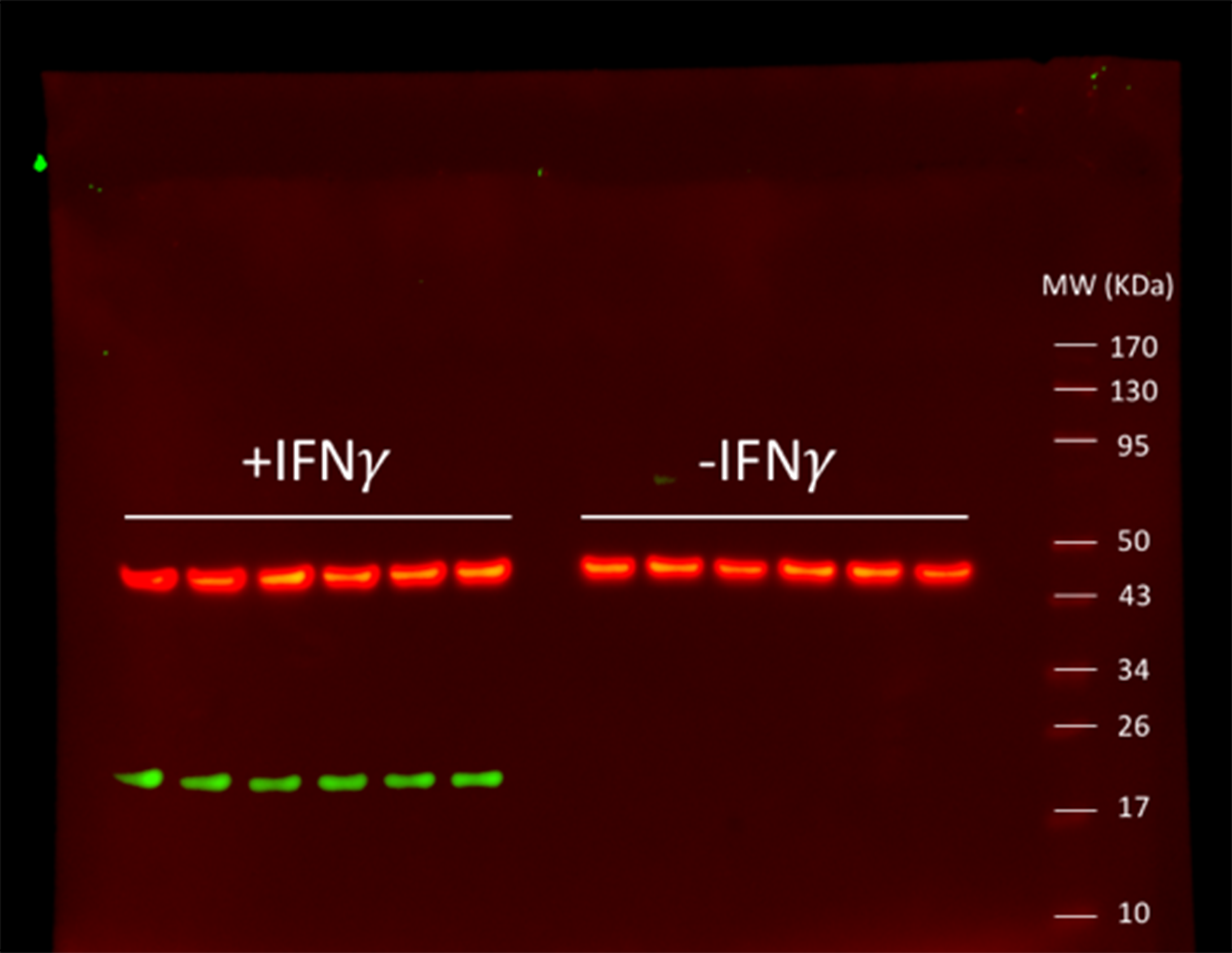


**Supplemental Figure 1. Exposing human MSCs to recombinant murine IFNγ *in vitro* increases IL-10 expression.** hMSCs were cultured in complete medium supplemented 250 U/mL recombinant murine IFN**γ** for 24 hr. MSCs were washed three times with phosphate buffered saline and lysed using a balanced solution containing 0.1% Triton X-100 and removed from the culture plate by scraping. Cell lysates were sonicated for 10 sec and centrifuged to remove insoluble material. Lysates were then prepared and analyzed using the protocols outlined in Experimental Methods for western blotting. The image shows the unaltered fluorescence imaging of hMSC lysates that were cultured with or without IFN**γ** (n=6 per group). A band corresponding to IL-10 at ~17 KDa (green) is present in the samples that were treated with IFN**γ**, but were absent in hMSCs that were cultured under normal conditions. Actin was probed as a loading control and appears at ~45 KDa (red).


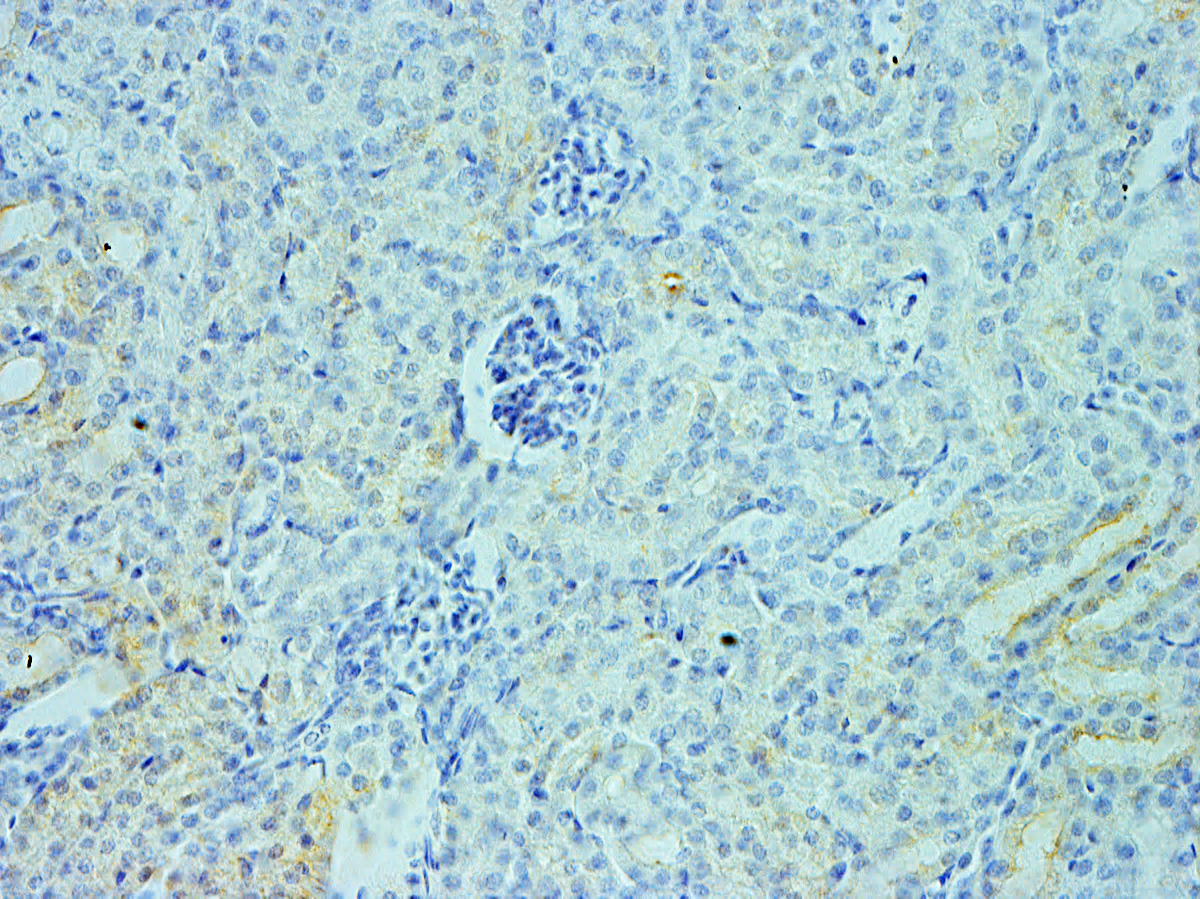

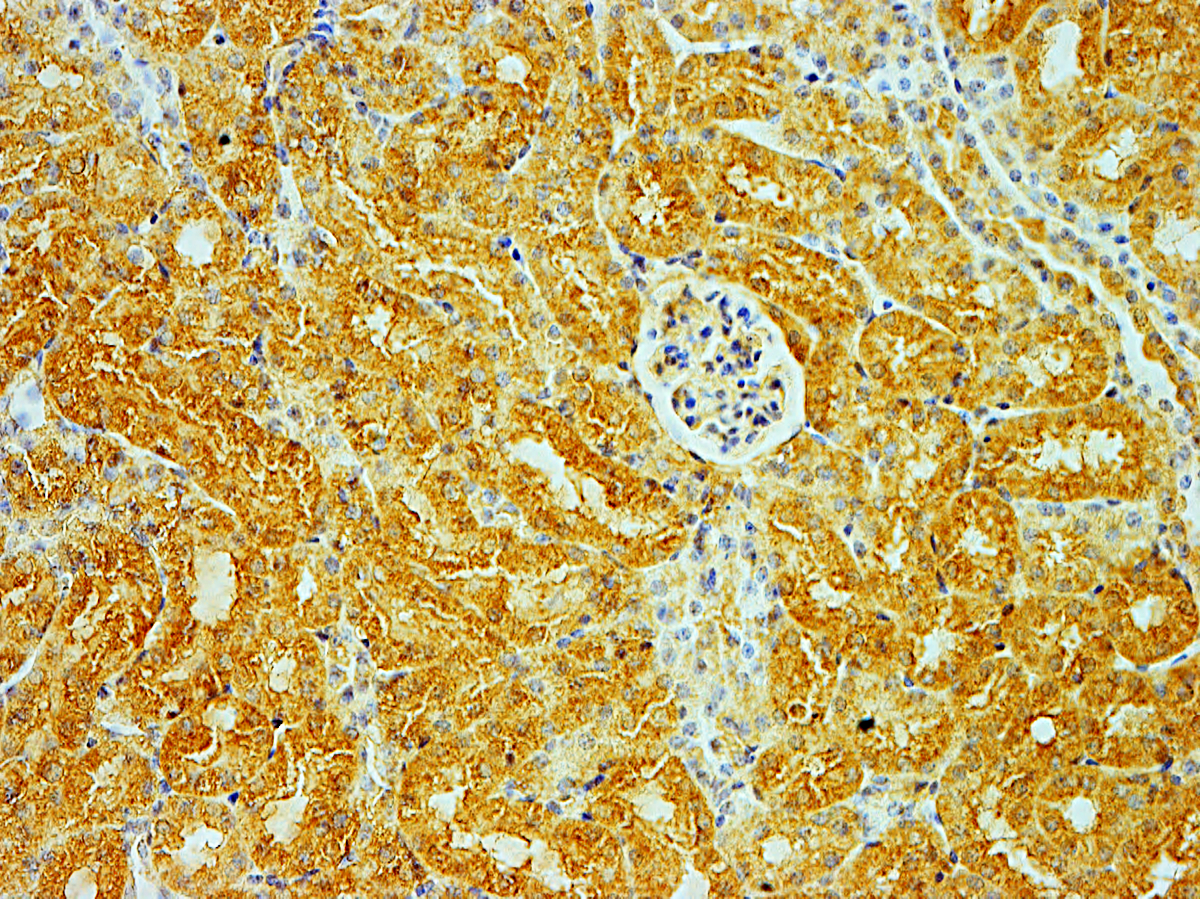


Untreated AKI

Normal Kidney

Normal Kidney

Untreated AKI

**
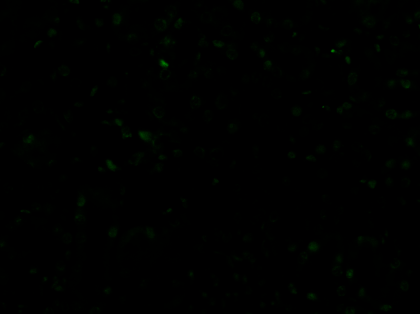
**
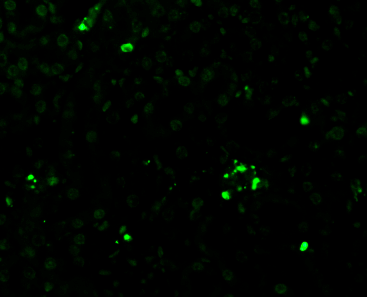


**Supplemental Figure 2. Comparison of KIM1 expression and TUNEL+ nuclei between normal kidneys and untreated AKI in C3H mice.** Top row is KIM1 immunostaining and bottom row is fluorescein-labeled DNA breaks.

**Supplemental Figure 3. pFUS at 4 MPa peak negative pressure (PNP) alone does not alter renal function during cisplatin-induced AKI.** C3H mice (n=6 per group) had AKI induced with cisplatin (15 mg/kg). Twenty-four hours post-cisplatin, one groups of mice received bilateral kidney pFUS while the other received a sham sonication (0W). Seventy-two hours later, mice were assayed for BUN and SCr levels. Similar to findings in [17], lowering pFUS PNP to 4 MPa does not influence AKI functional measurements alone.

**Supplemental Figure 4. pFUS increases renal IFNγ levels in C3H mice, but not in the IFNγ-KO mouse.** C3H (WT) or B6.129S7-*Ifngr1^tm1Agt^*/J (IFNγ-KO) mice (n=6 per group) had AKI induced by i.p. injection of cisplatin (15 mg/kg). Twenty-four hours after cisplatin treatment, mice were subjected to unilateral kidney pFUS so the contralateral kidney could serve as an internal control. Four hours post-pFUS, mice were euthanized and kidneys were harvested for analysis. Kidney homogenates (2 mg/mL) from treated and contralateral control kidneys were loaded into an IFNγ ELISA plate. pFUS increased IFNγ levels approximately 4 fold in C3H mice. IFNγ signal was near the limit-of-detection for the assay in both pFUS-treated and contralateral control kidneys from the B6.129S7-*Ifngr1^tm1Agt^*/J mice.

**
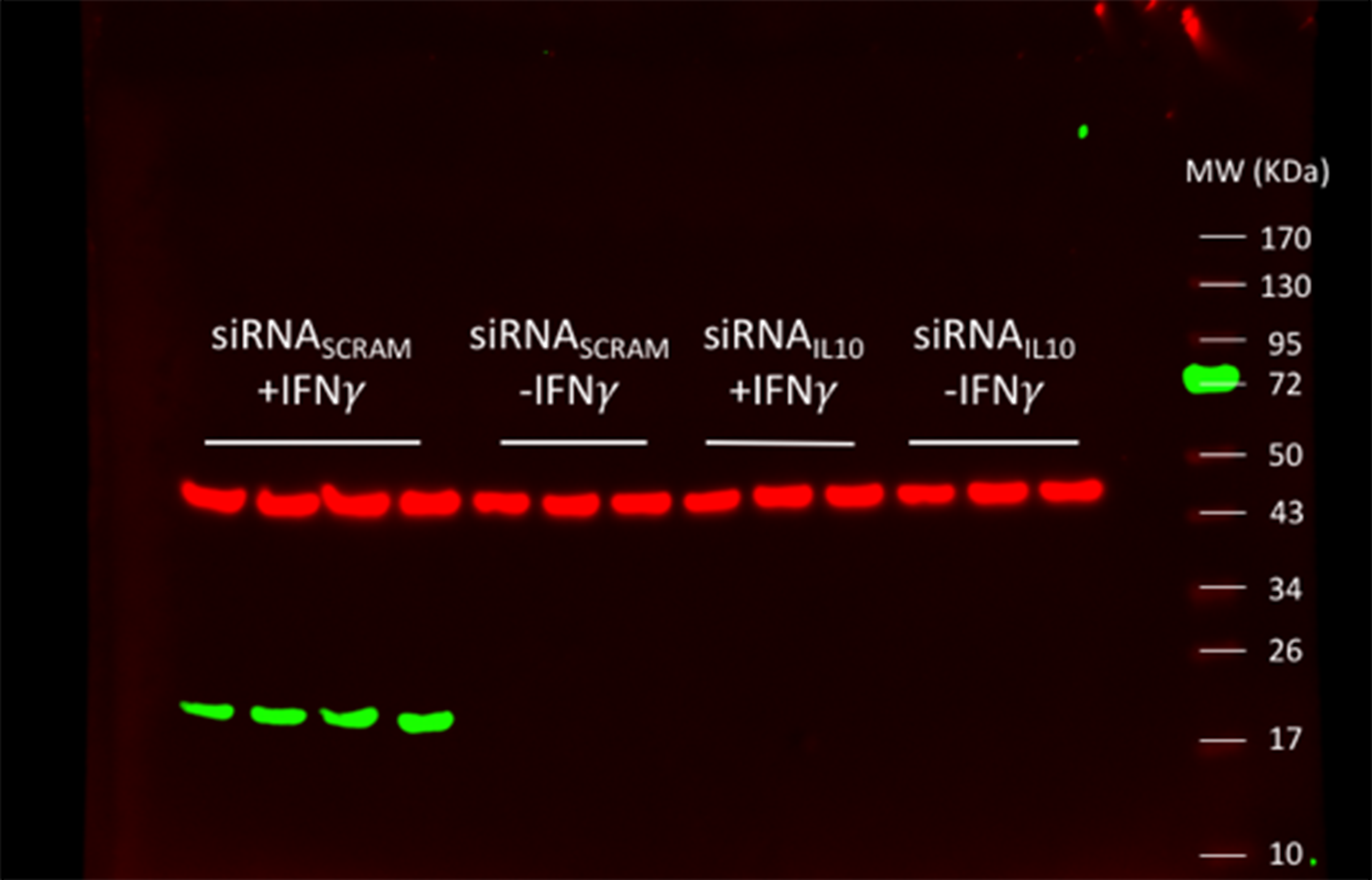
**

**Supplemental Figure 5. MSCs transfected with anti-IL-10 siRNA do not upregulate IL-10 following IFNγ pretreatment, but MSCs transfected with scrambled siRNA sequences do upregulate IL-10 following IFNγ pretreatment.** MSCs were transfected with siRNA against IL-10 (siRNA_IL10_) or control scrambled (siRNA_SCRAM_) as described in Materials and Methods. Both types of transfected MSCs were divided into groups that received IFNγ pretreatment or not. Cells were then harvested as described in Supplemental Figure 1 and western blotting was performed for human IL-10 as described in the Materials and Methods. MSCs that received the control siRNA sequences retained their ability to upregulate IL-10 (green bands ~17 KDa) following IFNγ pretreatment (n=4) compared to MSCs that were transfected with control siRNA but did not receive IFNγ pretreatment (n=3). However, transfecting MSCs with siRNA against IL-10 disrupted the ability for MSCs to upregulate IL-10 following IFNγ pretreatment (n=3). Actin loading controls (~45 KDa) appear as red bands.

**
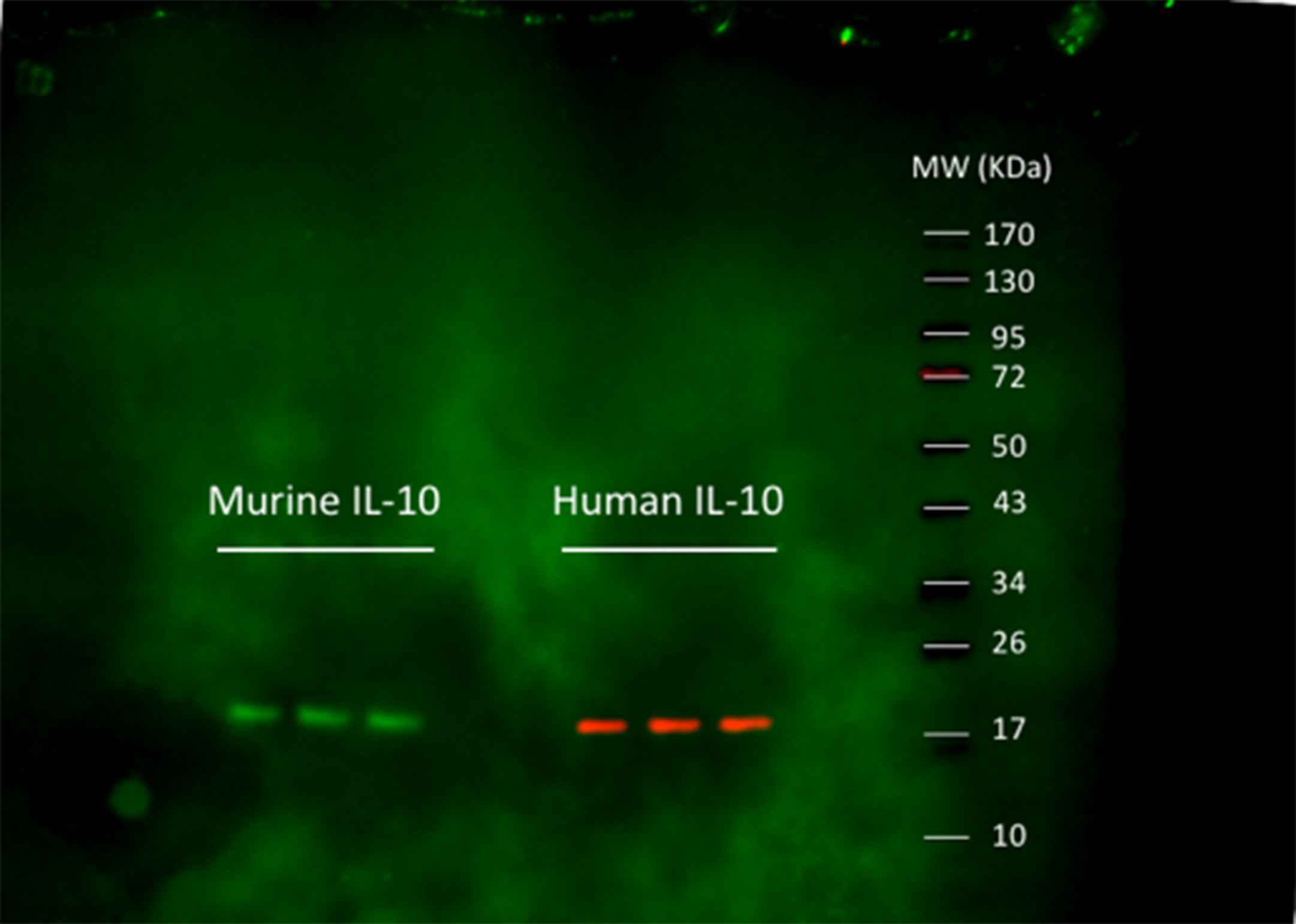
**

**Supplemental Figure 6. The detection antibody used for human IL-10 ELISAs does not cross react with murine IL-10.** Recombinant human IL-10 or recombinant murine IL-10 (2 ng/lane; n=3 per group) were electrophoresed and blotted for using human- or murine-IL-10-specific primary antibodies. Human IL-10 appears as red bands while murine IL-10 appears as the green bands.
